# Green Synthesis of AgNPs from *Celtis africana*: Biological and Catalytic Insights

**DOI:** 10.3390/nano15231821

**Published:** 2025-12-01

**Authors:** Amna N. Khan

**Affiliations:** Department of Chemistry, Faculty of Science, King Abdulaziz University, Jeddah 21589, Saudi Arabia; ankmohamad@kau.edu.sa

**Keywords:** *Celtis africana* aqueous leaf extract, green synthesis, silver nanoparticles, antimicrobial activity, cytotoxicity, antioxidant activity, catalytic activity

## Abstract

*Celtis africana,* a rare plant native to southwestern Saudi Arabia, was explored for the first time as a source for the green synthesis of silver nanoparticles (AgNPs). Catechol-bearing phenolic amides in the aqueous leaf extract acted as both reducing and capping agents, enabling eco-friendly AgNP fabrication. The synthesized AgNPs were characterized using SEM, TEM, XRD, UV-Vis, and FTIR, revealing predominantly spherical nanoparticles with an average size of 9.28 ± 0.11 nm, a face-centered cubic crystalline structure, and a pronounced surface plasmon resonance at 424 nm. HPLC analysis confirmed the presence of caffeoyltryamine in the extract, while UV-Vis and FTIR indicated its attachment to the AgNP surface. The AgNPs exhibited broad-spectrum antimicrobial activity against Gram-positive bacteria (*S. aureus*, MRSA and *E. faecalis*) and Gram-negative bacteria (*E. coli*, *K. pneumoniae*, *S. typhimurium*, and *P. aeruginosa*), as well as pathogenic fungi such as *C. albicans*, *C. glabrata*, *C. parapsilosis*, and *C. krusei* with performance comparable to or exceeding that of AgNPs from *Artemisia vulgaris*, *Moringa oleifera*, and *Nigella sativa*. The MIC and MBC values for *S. aureus*, MRSA, *E. coli*, and *S. typhimurium* were consistently 6.25 µg/mL and 25 µg/mL, respectively, reflecting strong inhibitory and bactericidal effects at low concentrations. MTT assays demonstrated selective cytotoxicity, showing higher viability in normal human skin fibroblasts (HSF) than in MCF-7 breast cancer cells. The AgNPs also displayed strong antioxidant activity (IC_50_ = 5.41 µg/mL, DPPH assay) and efficient catalytic reduction of 4-nitrophenol (4-NP) and methylene blue (MB), with rate constants of 0.0165 s^−1^ and 0.0047 s^−1^, respectively, exceeding most reported values. These findings identify *Celtis africana* as a promising source for eco-friendly AgNPs with strong antimicrobial, antioxidant, and catalytic properties for broad biological and environmental applications.

## 1. Introduction

*Celtis africana*, a species of the genus *Celtis* L., is indigenous to Saudi Arabia and Yemen and occurs sporadically along villages, valley banks, and the bottoms of rocky slopes [1,2,3]. Although numerous new plant species have been documented from Saudi Arabia in recent years, *Celtis africana* is reported here for the first time as a source for the phytogenic synthesis of AgNPs. In contrast to widely used regional medicinal plants such as *Artemisia vulgaris* [4,5,6], *Moringa oleifera* [7,8,9], and *Nigella sativa* [10,11,12], *Celtis africana* is not well represented in traditional Saudi folk medicine, likely due to its limited distribution and rarity [1]. Nonetheless, few studies have investigated its phytochemical content, antioxidant activity, and antimicrobial properties [2,3]. The presence of bioactive compounds such as flavonoids, tannins, polyphenols, and phenolic amides in *Celtis africana* suggests promising potential for its application in the phytogenic synthesis of AgNPs as a novel biomedical and catalytic agent. Plant-mediated AgNPs are known to combine the therapeutic benefits of medicinal plants with the efficacy and versatility of nanotechnology, resulting in enhanced, targeted, and stable biomedical and catalytic applications that surpass the capabilities of plant extracts alone [13,14]. Consequently, there has been a substantial rise in plant-based AgNPs synthesis over the past decades [15,16,17].

Gram-positive *S. aureus* is associated with a broad range of infections, from milk skin conditions to serious diseases such as osteomyelitis and septicemia, complicating clinical management [18]. MRSA is a multidrug-resistant variant of *S. aureus* that has developed resistance to β-lactam antibiotics such as methicillin, penicillin, and amoxicillin. It is a major cause of hospital-acquired infections and often necessitates the use of more potent or potentially toxic antibiotics [19]. *E. faecalis* is another opportunistic Gram-positive pathogen, commonly associated with infections such as urinary tract infections, endocarditis, and intra-abdominal infections. Its intrinsic resistance to antibiotics and ability to transfer resistance genes to other bacteria make it a key driver of antimicrobial resistance [20]. In contrast, *E. coli*, *K. pneumoniae*, *S. typhimurium*, and *P. aeruginosa* are Gram-negative bacteria, each with a distinct pathogenic profile. *E. coli* and *K. pneumoniae* are leading causes of urinary tract infections [21,22], *S. typhimurium* is primarily associated with gastrointestinal illness [23], and *P. aeruginosa* is notorious for hospital-acquired infections [24], especially in immunocompromised patients or those with open wounds or catheters. Each pathogen presents unique resistance patterns and clinical challenges, underscoring their importance in the context of escalating antimicrobial resistance.

Fungal infections, particularly those caused by *Candida* species, are difficult to diagnose early due to nonspecific symptoms and slow culture growth, often leading to delayed treatment and poor outcomes [25]. These pathogens frequently form biofilms on medical devices, enhancing resistance to antifungal agents and immune defenses. In immunocompromised individuals (e.g., cancer patients, transplant recipients, HIV/AIDS), such infections can be life-threatening. *C. albicans* is the most common cause of candidiasis [26], notable for its biofilm formation and morphological transition, contributing to both superficial and systemic infections like candidemia. *C. glabrata* shows high intrinsic resistance to fluconazole and often causes bloodstream and urinary tract infections [27]. *C. parapsilosis* is linked to catheter-related bloodstream infections, especially in neonates, and is associated with parenteral nutrition and device contamination [28]. *C. krusei* is inherently resistant to fluconazole and increasingly causes candidemia in immunosuppressed patients [29]. Together, these *Candida* species present significant clinical challenges due to their antifungal resistance, biofilm formation, and potential to cause severe infections. HSF cells, as non-cancerous primary cells, provide a reliable model for assessing biocompatibility and regenerative potential, whereas MCF-7 cells are widely employed to assess anticancer efficacy [30].

Oxidative stress-related diseases are caused by an imbalance between reactive oxygen species (ROS) production and the body’s ability to counteract them with antioxidants [31]. This imbalance leads to damage to DNA, proteins, lipids, and cellular structures, contributing to the onset and progression of various diseases [32]. Oxidative stress functions both as a cause and a consequence of inflammation, metabolic dysfunction, and cellular injury [33]. Addressing oxidative stress through antioxidant-based therapies and lifestyle changes is a growing focus in the prevention and treatment of many chronic conditions [34,35]. Wastewater contaminants encompass a wide variety of pollutants, including bacteria, fungi, heavy metal ions, dyes, and synthetic organic compounds [36]. Among these, dyes and persistent organic pollutants are especially difficult to degrade into less harmful or non-toxic forms through natural processes [37]. If left untreated, these contaminants can remain in aquatic systems, disrupt ecological balance, and eventually seep into groundwater sources [38]. This poses a significant health risk, as toxic substances may enter drinking water supplies undetected. Recently, AgNPs, particularly those synthesized through environmentally friendly methods using plant extracts, have emerged as a promising and sustainable solution for the efficient removal of such pollutants from wastewater [39,40].

Green synthesis of AgNPs follows the wet chemical reduction approach, wherein both reducing and capping agents are present in the aqueous phase. These agents successively reduce Ag^+^ ions to metallic silver (Ag^0^) and stabilize the AgNPs in solution [41]. During the formation of AgNPs, the nucleation and growth stages are critical in determining the final characteristics of the nanoparticles, including their size, shape, concentration, and degree of aggregation [42]. Phytochemicals in plant extracts play a pivotal role in both nucleation and growth. Compounds such as flavonoids, polyphenols, phenolic acids, and phenolic amides act as reducing agents by donating electrons to Ag^+^ ions, thereby converting them into Ag^0^ atoms that form the initial nanoparticle nuclei [43]. The strength and concentration of these reducing agents significantly affect the reduction rate; stronger or more abundant reducing agents accelerate nucleation, leading to the formation of a greater number of nuclei and consequently smaller, more uniformly sized nanoparticles [44]. Following nucleation, additional Ag^0^ atoms deposit onto these nuclei, contributing to the nanoparticles’ growth [42]. At this stage, phytochemicals bearing oxygen-containing functional groups, such as carboxylic acid and phenols/hydroxyls, adsorb onto the nanoparticle surfaces, functioning as capping agents that prevent aggregation and guide isotropic growth [43,44]. This steric or electrostatic stabilization helps regulate particle size and enhance colloidal stability [13,14]. Furthermore, the type and concentration of specific phytochemicals can influence the final shape of the nanoparticles, such as spheres, rods, cubes, or plates, by selectively binding to different crystal facets during growth [45]. Therefore, plant-mediated synthesis of AgNPs offers superior control over nucleation, growth kinetics, and morphological features compared to conventional chemical reduction methods, while also adhering to the principles of sustainability and green chemistry.

In this study, phytochemicals derived from the aqueous extract of *Celtis africana* leaves were employed for the green synthesis of AgNPs. The synthesized AgNPs were comprehensively characterized to assess SPR behavior, identify phytochemical constituents in both the extract and those adsorbed on the AgNPs surfaces, and examine their structural and morphological attributes. Characterization techniques included XRD, SEM, TEM, XRD, UV-Vis, and FTIR. The synthesis mechanism was interpreted based on a redox-mediated pathway. The phytofabricated AgNPs were subsequently evaluated for antimicrobial activity using the agar well diffusion method against selected bacterial and fungal strains. Their antioxidant capacity was assessed through the DPPH radical scavenging assay, and their catalytic performance was investigated by monitoring the reduction of 4-NP and MB in the presence of sodium borohydride (NaBH_4_).

## 2. Materials and Methods

Silver nitrate (AgNO_3_), DPPH radical, methanol, 4-NP, MB, and NaBH_4_ were all of analytical grade and obtained from Bayouni Trading Company (Merck distributor, Riyadh, Saudi Arabia). N-trans-caffeoyltyramine (98% purity) was supplied by Alpha roots (Jeddah, Saudi Arabia). All reagents were used as received, without any additional purification. Milli-Q water (Millipore SAS, Molsheim, France), recognized for its high purity and minimal ionic content, was used throughout the study for preparing all solutions and reaction media to ensure consistency and reduce the risk of contamination.

### 2.1. Collection and Identification of Celtis africana Leaves

**Scheme 1:**
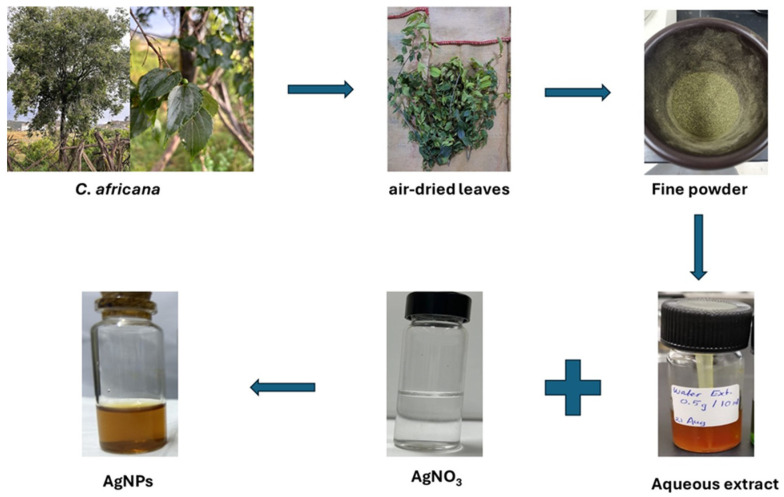
Schematic illustration of the collection of *Celtis africana* leaves, followed by air-drying, grinding, aqueous extract preparation, and the subsequent formation of AgNPs.

Fresh leaves of *Celtis africana* (Scheme 1) were hand-collected from Wadi Shura, located in Baljurashi city, Al-Baha, Kingdom of Saura Arabia. The plant was identified by Dr. Faten Zubair Flimban, a plant taxonomist in the Division of Botany, Department of Biology, Faculty of Science, King Abdulaziz University, Jeddah, Saudi Arabia. Leaves were rinsed with deionized water, air-dried at room temperature, and stored in sealed plastic bags until use. To ensure reproducibility, leaves were collected twice over a 6–8-month period, processed into powder, and used consistently. AgNPs (SPR band at 424 nm) were synthesized regularly, and no significant variation was observed in UV–Visible spectra or visual appearance, indicating stable phenolic content and reproducible synthesis.

### 2.2. Preparation of Aqueous Celtis africana Leaf Extract

To prepare the aqueous extract, approximately 0.5 g of *Celtis africana* leaves was soaked in 10 mL of Milli-Q water at room temperature for 24 h to facilitate the extraction of bioactive phytochemicals [13,14]. The resulting extract, with a concentration of 50 mg/mL, was considered sufficient to supply the reducing and stabilizing agents necessary for the synthesis of AgNPs. Following extraction, the mixture was centrifuged (Virtuose® PRP centrifuge system, Langfang Baimu Medical Devices Co., Ltd., Langfang, China) at 5000 rpm for 5–10 min to remove solid residues. The supernatant, which displayed a characteristic burnt orange color (Scheme 1), was carefully collected and stored at 4 °C for later use in AgNPs synthesis.

### 2.3. Synthesis of AgNPs

AgNPs were synthesized by reacting the aqueous extract of *Celtis africana* leaves with AgNO_3_ in Milli-Q water. In a typical procedure, 100 µL of *Celtis africana* extract (50 mg/mL) was mixed with 100 µL of 0.1 M AgNO_3_ solution (containing 10.787 mg/mL of Ag^+^) in 5 mL of Milli-Q water [13,14]. This resulted in final concentrations of 1000 µg/mL for the plant extract and 215.7 µg/mL for Ag^+^ ions in the reaction mixture. The solution was left undisturbed at room temperature for 12 h. A visible color change from light brown to dark brown indicated the successful formation of AgNPs (Scheme 1). The reaction progress and nanoparticle formation were monitored using UV–Visible spectroscopy, revealing a prominent SPR band at 424 nm.

Based on 1:1 stoichiometric reduction of Ag^+^ to Ag^0^, the final concentration of AgNPs was estimated to be 215.7 µg/mL. To investigate dose-dependent antimicrobial activity, additional concentrations were prepared by varying the volumes of AgNO_3_ and plant extract accordingly. The entire synthesis process was conducted under ambient conditions, highlighting the simplicity, adaptability, and potential scalability of this green synthesis approach.

### 2.4. Structural and Morphological Characterization

The SPR properties of the synthesized colloidal AgNPs were analyzed directly using a Cary 60 UV–Visible spectrophotometer (Agilent Technology, Santa Clara, CA, USA). The phytochemicals present in the aqueous extract of *Celtis africana* leaves and those adsorbed onto the surface of AgNPs were characterized using UV–Visible and FTIR spectroscopy (Cary 630, Agilent Technology, Santa Clara, CA, USA) over the range of 4000–400 cm^−1^. The aqueous extract was analyzed directly by UV–Visible, whereas for FTIR analysis, both the AgNPs and the aqueous extract were drop-cast onto glass slides and air-dried prior to measurement. HPLC analysis of the aqueous extract of *Celtis africana* leaves was performed using an SPD-20A HPLC system (Shimadzu, Kyoto, Japan) with an RP-C18 column and a UV detector at 254 nm. The aqueous leaf extract of *Celtis Africana* was diluted with the mobile phase (acetonitrile/acetic acid, 50:50 *v*/*v*) immediately before injection into the HPLC system. The crystalline structure of the AgNPs was examined using an Ultima IV multipurpose X-ray diffractometer (Rigaku, Akishima, Japan), employing AgNPs coated on a glass slide as the sample. The morphology and aggregation state of the synthesized AgNPs were investigated using SEM (JEOL, SM-IT700HR, Tokyo, Japan), while particle size was determined by TEM (JEOL, JEM-2100f, Peabody, MA, USA). Samples for both SEM and TEM were prepared by drop-casting the colloidal solution of AgNPs onto a silicon wafer and allowing it to dry at room temperature. The surface charge (zeta potential) of the AgNPs was determined using a Zetasizer Nano ZS (Malvern Instruments, Malvern, UK).

### 2.5. Antimicrobial Activity

The antibacterial activity of *Celtis africana* extract-mediated AgNPs was assessed against both Gram-positive bacteria (*S. aureus*, MRSA, and *E. faecalis*) and Gram-negative bacteria (*E. coli*, *K. pneumoniae*, *S. typhimurium*, and *P. aeruginosa*) using the agar well diffusion method [13,14]. All bacterial strains were obtained from the King Fahad Medical Research Center (KFMRC), Jeddah, Saudi Arabia. Fresh bacterial suspensions were prepared according to standard microbiological protocols and adjusted to a 0.5 McFarland turbidity standard. For the assay, 0.1 mL of each bacterial suspension was evenly spread on nutrient agar plates, after which 6 mm wells were punched using a sterile cork borer. Each well was then filled with 100 µL of AgNPs (215.7 µg/mL). The plates were then incubated at 37 °C for 24 h, and the antibacterial activity was assessed by measuring the diameter of the inhibition zones. Tetracycline (40 µg/mL) was used as a positive control, while sterile distilled water served as a negative control.

For antifungal activity assessment, 0.1 mL of each fungal suspension of *C. albicans*, *C. glabrata*, *C. parapsilosis*, and *C. krusei* was uniformly spread onto Potato Dextrose agar plates [13,14]. Wells with a diameter of 6 mm were aseptically punched using a sterile cork borer and subsequently filled with 100 µL of AgNPs (215.7 µg/mL). The plates were then incubated at 37 °C for 24 h. After incubation, the diameter of the zones of inhibition was measured to assess antifungal activity. Fluconazole (25 µg/mL) was used as the positive control, while sterile distilled water served as the negative control.

### 2.6. MIC and MBC

The minimum inhibitory concentration (MIC) of the synthesized AgNPs was determined using the broth microdilution method in 96-well microtiter plates [13,14]. Fresh bacterial cultures of *S. aureus*, MRSA, *E. coli*, and *S. typhimurium*, representing both Gram-positive and Gram-negative bacteria, were prepared and adjusted to match the turbidity of a 0.5 McFarland standard. Each 96-well plate consisted of 12 columns. AgNPs were serially diluted across columns 1 to 10 using a two-fold dilution method to achieve final concentrations of 100, 50, 25, 12.5, 6.25, 3.12, 1.56, 0.78, 0.39, and 0.195 µg/mL, respectively. Each well was then inoculated with 100 µL of the respective bacterial suspension. Column 11 served as the positive control (bacterial culture without AgNPs), while column 12 was used as the negative control (nutrient broth only). In total, four plates were prepared, one for each bacterial strain. The plates were incubated at 37 °C for 22 h. After incubation, 20 µL of 0.2% (*w*/*v*) resazurin solution was added to each well as an indicator of bacterial viability. The plates were then incubated for an additional 4 h at 37 °C. The MIC was defined as the lowest concentration of AgNPs that inhibited visible bacterial growth, indicated by the retention of the blue color of the resazurin dye (no color change).

The minimum bactericidal concentration (MBC) was determined using the same 96-well microtiter plate employed for the MIC assay [13,14]. From wells showing no visible color change, 20 µL of the mixture was aseptically transferred and spread onto agar plates, followed by incubation at 37 °C for 24 h. The MBC was defined as the lowest concentration of the sample at which no bacterial colonies were observed, indicating complete bactericidal activity.

To determine the IC_50_ values of AgNPs and tetracycline, a series of concentrations (100, 50, 25, 12.5, 6.25, 3.12, 1.56, 0.78, 0.39, and 0.195 µg/mL) was individually applied to bacterial cultures in a 96-well microtiter plate and incubated at 37 °C for 24 h. Absorbance was then measured to assess bacterial growth inhibition. The recorded absorbance values were plotted against the respective concentrations, and IC_50_ values were estimated using linear regression by substituting an absorbance value of 0.5, corresponding to 50% inhibition.

### 2.7. Bacterial Growth Behavior

The growth behavior of *S. aureus*, MRSA, *E. coli*, and *S. typhimurium* bacteria was monitored over time to evaluate the effect of AgNPs on bacterial proliferation [13,14]. Four 96-well plates were prepared as described in the MIC assay. AgNPs concentrations across columns 1 to 10 were maintained at 100, 50, 25, 12.5, 6.25, 3.12, 1.56, 0.78, 0.39, and 0.195 µg/mL, respectively. To assess bacterial growth dynamics, absorbance at 600 nm (OD_600_) was measured at regular time intervals using a UV–Visible spectrophotometer. The optical density (OD_600_) readings were corrected by subtracting the absorbance at time zero. Growth curves were plotted for each bacterial strain to illustrate changes in viability and proliferation over time in response to varying AgNPs concentrations.

### 2.8. Cytotoxicity Assay

HSF and MCF-7 cell lines were maintained under sterile conditions according to ATCC guidelines in DMEM supplemented with 10% FBS and 1% penicillin–streptomycin at 37 °C in a humidified 5% CO_2_ incubator. Cells were routinely sub-cultured and maintained under standard conditions until sub-confluence. The in vitro cytotoxicity of AgNPs was evaluated using the standard MTT assay [8,30]. Cellular metabolic activity was used as an indicator of cytotoxicity, since the reduction of MTT to purple formazan crystals is directly proportional to the number of metabolically active cells. The assay was performed in a 96-well plate, with each well seeded with 1 × 10^4^ viable cells and incubated at 37 °C in 5% CO_2_ for 20 h. AgNPs (100 µg/mL) were added to the wells and incubated for 24 h, while untreated cells served as controls. Subsequently, 10 µL of MTT solution (5 mg/mL in PBS) was added to each well and incubated for 4 h. Formazan crystals were solubilized in 100 µL of DMSO, and absorbance was measured at 570 nm using an ELISA plate reader. Cell viability was expressed as the percentage ratio of the absorbance of treated to untreated cells × 100. Morphological changes were examined under an inverted light microscope (Nikon, Tokyo, Japan) at 10× magnification.

### 2.9. Antioxidant Efficacy

The antioxidant activity of the synthesized AgNPs was evaluated using the DPPH free radical scavenging assay [13,14]. A stock solution of DPPH (4.3 mg/mL in 3 mL methanol) was prepared and subsequently diluted to obtain a working solution with a final concentration of 10 µg/mL. Different concentrations of AgNPs were then mixed with DPPH solution (10 µg/mL). The mixtures were incubated in the dark for 30 min to facilitate the reaction. Following incubation, absorbance was measured at 517 nm using a UV–Visible spectrophotometer, and readings were corrected against a methanol blank. The percentage of DPPH radical scavenging activity was calculated using the following formula:

Scavenging activity (%) (Abs_blank_ − Abs_sample_/Abs_blank_) × 100

### 2.10. Catalytic Activity

The catalytic activity of the synthesized AgNPs was assessed by monitoring the reduction of 4-NP and MB. In a typical procedure, 100 µL of the AgNPs suspension was separately added to 10 mL of 20 µg/mL aqueous solutions of 4-NP and MB. NaBH_4_ (2 mL, 0.1 M) was used in excess to ensure that the reaction followed pseudo-first-order (ln*A_t_* = −*k*_app_ *t* + ln*A*_0_) kinetics with respect to the nitrophenol or dye [13,14]. The progress of the reactions was monitored at regular intervals (ranging from 5 to 120 min, with progressively increasing measurement intervals) by recording absorption spectra using a UV–Visible spectrophotometer. Reaction rates were determined from the time-dependent changes in absorbance, providing a measure of the catalytic efficiency of the AgNPs for the reduction of 4-NP and MB. Furthermore, an industrial wastewater sample (pH ≈ 7.8, electrical conductivity 1800–2500 µS/cm) containing primarily organic dyes and phenolic compounds was analyzed for the presence of 4-NP and MB, and catalytic reduction experiments were subsequently performed under similar conditions.

### 2.11. Statistical Analysis

All experiments were performed in triplicate, and the results are presented as the mean ± standard deviation (SD). One-way analysis of variance (ANOVA) was performed using OriginPro 9 (OriginLab, Northampton, MA, USA) to compare the means of different groups, and statistical significance was considered at *p* < 0.05.

## 3. Results and Discussion

### 3.1. SEM Analysis

**Figure 1:**
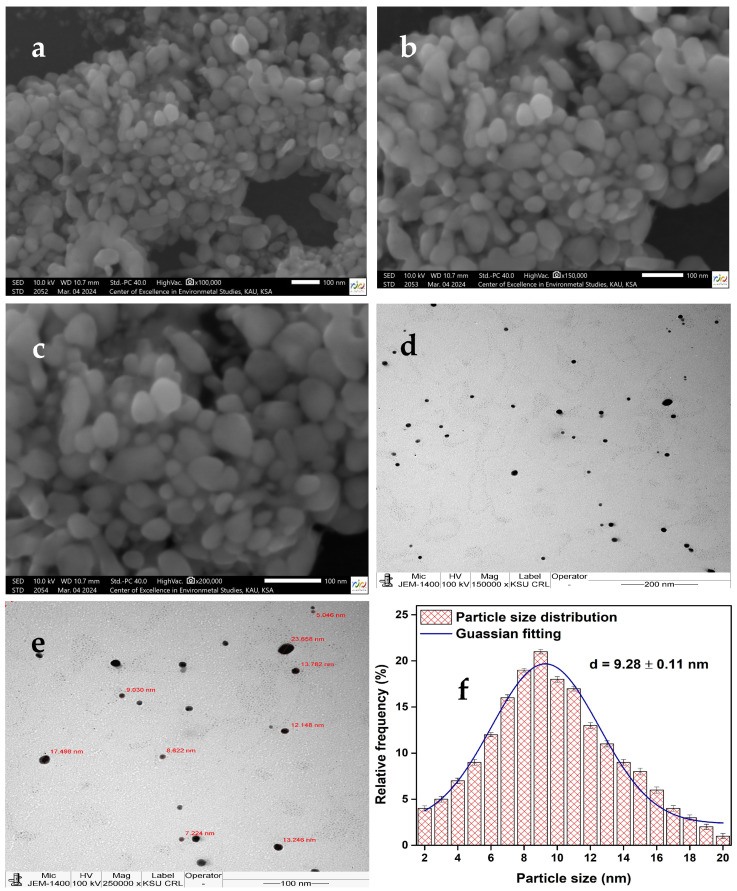
SEM images of AgNPs at magnifications of (**a**) 100,000, (**b**) 150,000, and (**c**) 250,000×; TEM images at (**d**) 150,000× and (**e**) 250,000×; and (**f**) particle size distribution.

The morphology, uniformity, and aggregation behavior of AgNPs synthesized using the aqueous leaf extract of Celtis africana were examined using SEM. For analysis, the AgNPs solution was drop-cast onto a silicon wafer and allowed to air-dry. SEM images were captured at magnifications of (a) 100,000×, (b) 150,000×, and (c) 250,000×, as shown in Figure 1a–c. The phytochemicals present in the Celtis africana aqueous extract appeared to play a crucial role in mediating the formation of densely packed AgNPs, with most particles preserving their individual shapes and avoiding significant bulk aggregation. Nevertheless, a certain degree of aggregation was observed, particularly among bigger particles forming rod-like or elongated structures. The AgNPs exhibited a wide range of particle sizes, varying from as small as 10 nm to as large as 50 nm. Smaller particles were predominantly oval-shaped, whereas larger ones exhibited diverse and irregular morphologies, including tetrahedral and hexagonal forms [42]. This moderate heterogeneity in particle size and shape may enhance antimicrobial performance by enabling multiple modes of interaction with microbial cell surfaces. Furthermore, the phytochemicals capping the AgNPs likely contributed not only to shape control but also to mediating close-range interactions with microbial membranes, potentially facilitating better adhesion and efficacy.

### 3.2. TEM Particle Size Distribution

TEM analysis was employed to determine the size distribution and average diameter of the synthesized AgNPs. Samples were prepared by drop-casting the AgNPs solution onto a silicon wafer and air-drying, following a procedure similar to that used for SEM. TEM images were captured at magnifications of 150,000× and 250,000×, as shown in Figure 1d,e. While SEM analysis predominantly revealed oval and irregular morphologies, TEM provided higher-resolution insights confirming that the AgNPs were mostly spherical with uniform morphology and minimal structural irregularities. The majority of the AgNPs fell within a 5–20 nm size range, with only a small proportion of larger particles, offering a narrower distribution than that observed in SEM. The particle size distribution, presented in Figure 1f, was fitted with a Gaussian fitting, yielding a mean particle size of approximately 9.28 ± 0.11 nm. This narrow size range and ultrasmall average diameter are particularly significant, as smaller nanoparticles offer a high surface-area-to-volume ratio, which can enhance interaction with microbial membranes and improve antimicrobial performance. The TEM results indicate that the aqueous extract of Celtis africana leaves effectively facilitated the phytogenic synthesis of uniformly dispersed, spherical, and sub-10 nm AgNPs. To the best of my knowledge, this study represents the first report of AgNPs synthesized using Celtis africana, highlighting a novel botanical resource for biogenic nanoparticles fabrication.

### 3.3. XRD Analysis

**Figure 2:**
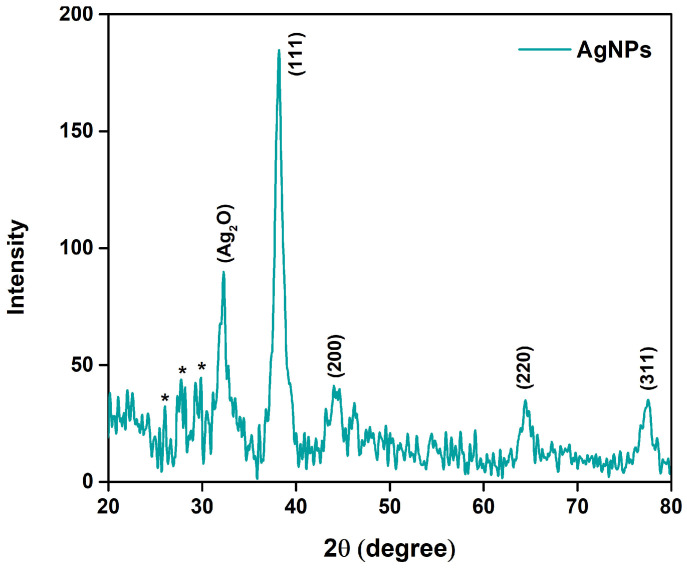
XRD pattern of the synthesized AgNPs showing characteristic diffraction peaks. Peaks marked with an asterisk (*) correspond to bio-organic residues responsible for capping the nanoparticles.

The crystalline nature of the synthesized AgNPs was examined using XRD over a 2*θ* range of 20°–80°, and the resulting diffraction pattern is presented in Figure 2. Four prominent diffraction peaks appeared at 2*θ* values of 38.18°, 44.20°, 64.43°, and 77.40°, corresponding to the (111), (200), (220), and (311) crystallographic planes of fcc Ag, respectively. These reflections confirmed the crystalline and fcc nature of the AgNPs, in agreement with the JCPDS standard powder diffraction card (silver file No. 04-0783) [13,14,46,47]. A peak at 2*θ* = 32.34° was also detected, which may be attributed to unreduced AgNO_3_ or the possible formation of silver oxide (Ag_2_O) due to partial surface oxidation of the AgNPs during sample preparation. In addition, several low-intensity peaks marked with an asterisk below 2*θ* = 30° were observed, likely originating from crystalline phytochemical constituents of the *Celtis africana* extract. These signals suggest the presence of bio-organic residues capping or interacting with the surfaces of the AgNPs [13,14]. Overall, the XRD analysis confirmed the successful formation of crystalline AgNPs and indicated a possible contribution of plant-derived biomolecules to their structural characteristics and stability.

### 3.4. UV–Visible Spectroscopy of Plant Extract and AgNPs

**Figure 3:**
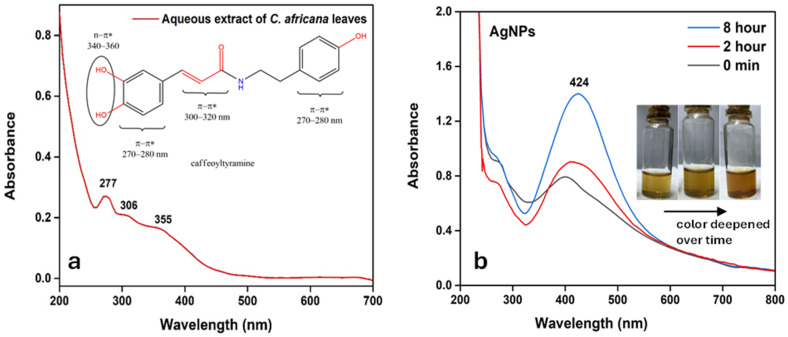
(**a**) UV–Visible spectrum of the aqueous extract of *Celtis africana* leaves; (**b**) UV–Visible spectra of AgNPs recorded at different time intervals.

UV–Visible spectroscopy was employed to investigate both the phytochemical composition of the aqueous *Celtis africana* leaf extract and the formation kinetics of the synthesized AgNPs. The spectrum of the crude extract (Figure 3a) exhibited three distinct absorption peaks at 277, 306, and 355 nm. According to literature, these bands are characteristics of phenolic amide compounds [48,49,50,51]. The peak at 277 m corresponds to π-π* transition within aromatic rings, while the band at 306 nm is associated with π-π* transition in systems with extended conjugation, such as C=C-C=O moieties, potentially influenced by amide resonance. The absorption at 355 nm is particularly indicative of catechol groups (ortho-dihydroxybenzene moieties), known for their strong light absorption and redox potential. The higher-wavelength transition indicates a notable presence of catechol-bearing phenolic amides, such as caffeoyltyramine [2,3], unlike simpler anlages like *p*-coumaroyltyramine, which lack the hydroxyl group needed for catechol formation.

This interpretation aligns with reports on the phytochemistry of *Celtis africana*, which document various phenolic amides [2,3]. Caffeoyltyramine, in particular, possesses an ortho-dihydroxyl configuration that facilitates efficient electron delocalization and promotes favorable oxidation to o-quinones [13]. These redox-active intermediates play a critical role in reducing Ag^+^ ions to metallic Ag^0^. Consequently, catechol-bearing phenolic amides are likely key contributors to the bioreduction and stabilization of uniformly dispersed AgNPs.

Figure 3b shows the UV–Visible spectra of the AgNPs recorded at various time intervals, with the inset illustrating the corresponding visual color change from light brown to dark brown upon the addition of AgNO_3_ to the plant extract. This progressive darkening of the solution visually confirmed the formation of AgNPs. Simultaneously, a SPR peak emerged at 424 nm, characteristic of AgNPs as widely reported in the literature [13,14]. The gradual increase in SPR intensity indicated ongoing nanoparticle nucleation and growth. After 8 h, the SPR signal stabilized, suggesting the completion of nanoparticle formation and production of well-dispersed, matured AgNPs. The synthesized AgNPs demonstrated notable colloidal stability, maintaining their SPR characteristics with minimal spectral changes for up to 7 days, indicative of negligible aggregation or degradation. After this period, spectral shifts, color changes, and increased turbidity were observed, suggesting partial aggregation; nonetheless, the AgNPs remained detectable in solution for up to 30 days. These results highlight their stability and suitability for antimicrobial, antioxidant, and catalytic applications. Zeta potential measurements (−22.2 ± 0.8 mV) further confirm moderate colloidal stability. The negative surface charge also suggests that the AgNPs are capped with negatively charged functional groups, such as o-quinone. Notably, 100 µL of 50 mg/mL *Celtis africana* leaf extract produced 215.6 µg/mL of AgNPs when mixed with 100 µL of 0.1 M AgNO3, yielding significantly more than extracts from *Artemisia vulgaris*, *Moringa oleifera*, and *Nigella sativa* [4,5,6,7,8,9,10]. Furthermore, although the characteristic catechol-related peak was absent in the UV–Visible spectra of the AgNPs, likely due to oxidation during the reduction process, the peak corresponding to the π-π* transition within aromatic ring systems at 277 nm remained evident. This suggests that some aromatic or phenolic residues persisted on the AgNPs surface, possibly contributing to their stabilization.

### 3.5. FTIR Spectral Analysis

**Figure 4:**
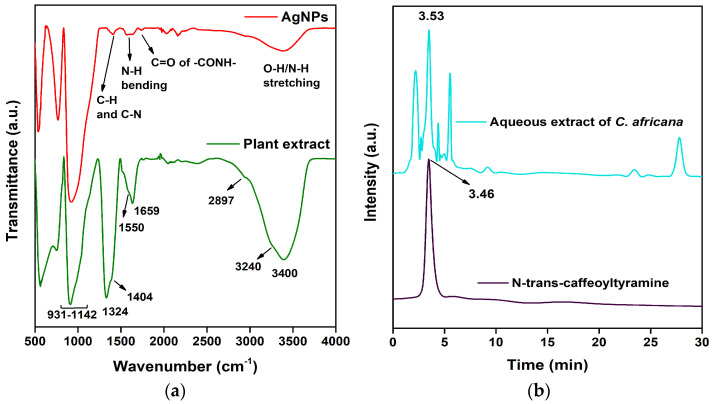
(**a**) FTIR spectra of the aqueous extract of *Celtis africana* leaves and synthesized AgNPs; (**b**) HPLC chromatogram of the extract showing phenolic amide-like compounds.

To further verify the presence of catechol-containing phenolic amides, FTIR spectroscopy was conducted on the aqueous extract of *Celtis africana* leaves. The spectrum, recorded over the range 4000–400 cm^−1^ (Figure 4a), showed several characteristic absorption bands corresponding to key functional groups present in phenolic amides such as caffeoyltyramine. These compounds typically exhibit broad bands between 3200–3500 cm^−1^ due to overlapping phenolic O-H and amide N-H stretching vibrations. Consistent with this, the FTIR spectrum displayed a broad, intense band centered at 3400 cm^−1^ with a shoulder at 3240 cm^−1^, indicative of the combined O-H and N-H stretching modes. A broader shoulder observed at 2897 cm^−1^ was attributed to C-H stretching vibrations from aliphatic chains. The amide C=O stretching vibration, a hallmark of the -CONH- linkage, was clearly observed at 1659 cm^−1^. The amide band, corresponding to N-H bending, appeared as a shoulder at 1550 cm^−1^, a region diagnostic for amide groups. Additional bands were identified at 1404 cm^−1^ (C-H bending overlapped with C-N stretching) and a sharper peak at 1324 cm^−1^, attributed to C-N stretching vibrations. The C-C and C-O stretching vibrations were detected as broad overlapping features in the region 1142–931 cm^−1^. These spectral characteristics align closely with reported FTIR data for phenolic amides containing catechol functionalities [52,53,54], although specific spectra for caffeoyltyramine are limited in the literature.

The FTIR spectrum of the synthesized AgNPs (Figure 4a) revealed a marked decrease in the intensity of the broad O-H/N-H stretching band, while its position remained nearly unchanged. This attenuation suggests the involvement of catechol O-H groups in the reduction of Ag^+^ ions to Ag^0^, likely through oxidation to o-quinone. The amide N-H bending peak at ~1550 cm^−1^ remained with comparable intensity, suggesting that the nitrogen was not directly involved in the reduction process. The C=O stretching of the -CONH- group and C-N stretching (partially masked by C-H bending), along with C-C and C-O regions, remained largely unaffected, indicating that the amide functional group was preserved. These findings support the conclusion that catechol groups played a central role in reducing Ag^+^ ions, while the amide moiety likely contributed to AgNPs stabilization through capping. The persistence of key amide-related bands suggests that the phenolic amide may function as both a reducing and stabilizing agent, catechol undergoing oxidation, and amide facilitates surface coordination.

### 3.6. HPLC Analysis of Celtis africana Leaf Extract

HPLC was employed to analyze the aqueous leaf extract of *Celtis africana* using a reverse-phase C-18 column and a mobile phase of acetonitrile/acetic acid (50:50) in order to identify phenolic amide-like compounds potentially contributing to the plant’s bioactivity [13,14]. The chromatogram (Figure 4b) showed multiple peaks between retention times 2–6 min, suggesting the presence of structurally related compounds with similar functional groups. To confirm the presence of caffeoyltyramine, a standard was run under identical conditions, yielding a peak at 3.46 min. A closely matching peak at 3.52 min in the extract confirmed its presence. The retention time and peak shape suggest that caffeoyltyramine is one of the major phenolic amides in the extract, contributing to its reducing and stabilizing properties during AgNPs synthesis.

### 3.7. Synthesis Mechanism of AgNPs

**Scheme 2:**
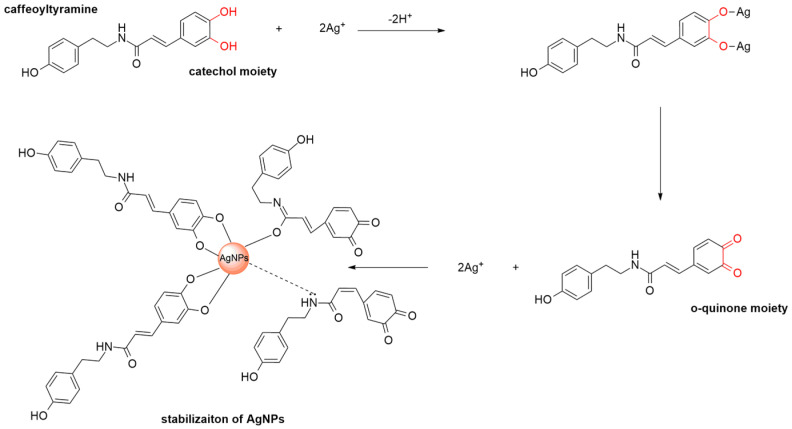
Synthesis and stabilization of AgNPs.

The mechanism underlying the phytogenic synthesis of AgNPs using the aqueous extract of *Celtis africana* leaves was elucidated based on phytochemical evidence and spectroscopic analysis. *Celtis africana* is known to be particularly rich in phenolic amides, especially bearing catechol moieties such as caffeoyltyramine. UV–Visible, FTIR, and HPLC analysis all support the involvement of these catechol-bearing compounds in the reduction of Ag^+^ ions to metallic Ag^0^, likely providing greater reducing capacity than their monophenolic analogs. A plausible reduction mechanism is illustrated in Scheme 2. Initially, the two hydroxyl groups of the catechol moiety undergo deprotonation, facilitating the formation of a coordination complex with Ag^+^ ions. This interaction promotes electron transfer, reducing Ag^+^ to metallic Ag^0^ while concurrently oxidizing the catechol to o-quinone. The resulting o-quinone species, along with preserved amide functionalities, adsorb onto the surface of the AgNPs via electrostatic interactions and hydrogen bonding [13]. These interactions contribute to the stabilization of the AgNPs, as indicated by a zeta potential of −22.2 ± 0.8 mV, and promote their uniform dispersion in the colloidal medium [14]. This dual functionality, reduction by catechol and capping through both the oxidized o-quinone and amide groups, highlights the key role of catechol-bearing phenolic amides in both the nucleation and stabilization phases of AgNPs formation.

### 3.8. Antimicrobial Activity Evaluation

**Figure 5:**
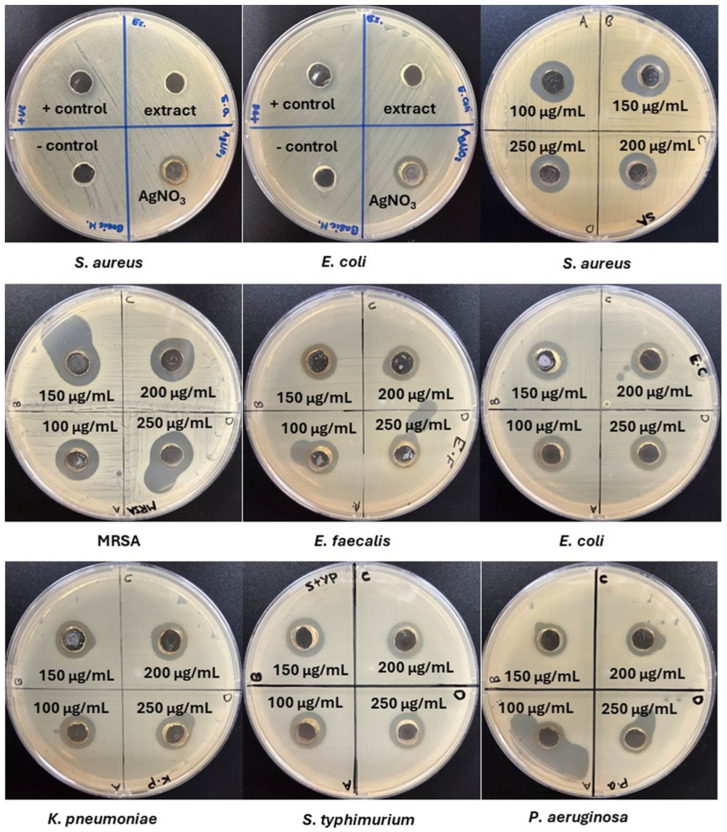
Agar well diffusion results showing the antibacterial activity of the aqueous extract of *Celtis africana* and AgNO_3_ against *S. aureus* and *E. coli* (representative strains), and of AgNPs at different concentrations against *S. aureus*, MRSA, *E. faecalis*, *E. coli*, *K. pneumoniae*, *S. typhimurium*, and *P. aeruginosa*.

The antibacterial activity of the aqueous extract of *Celtis africana* leaves, AgNO_3_, and the synthesized AgNPs was assessed against Gram-positive (*S. aureus*, MRSA, and *E. faecalis*) and Gram-negative (*E. coli*, *K. pneumoniae*, *S. typhimurium*, and *P. aeruginosa*) bacteria using the agar well diffusion assay at a concentration of 100 µg/mL. As shown in Figure 5 and detailed in Table 1, the plant extract showed no detectable inhibition zones against any of the tested strains, likely due to insufficient phytochemical content to exert direct antibacterial effects, despite being adequate for AgNPs synthesis. In contrast, AgNO_3_ exhibited measurable antibacterial activity against all strains, primarily due to the release of Ag^+^ ions, which disrupt membrane and cellular components such as thiol-containing enzymes and nucleic acids. However, this effect is limited in scope and duration. The synthesized AgNPs outperformed both the plant extract and AgNO_3_, producing large zones of inhibition across all strains. This enhanced efficacy is attributed to the multifaceted antibacterial mechanisms of AgNPs, including increased microbial contact due to high surface area, membrane disruption, sustained Ag^+^ ions release, and ROS generation [13,14,54,55]. Collectively, these findings confirm the broad-spectrum antibacterial potential of phytogenic AgNPs and underscore their promise for future antimicrobial applications.

The antibacterial efficacy of the synthesized AgNPs was also compared with tetracycline, as presented in Table 1. Except for *P. aeruginosa*, the zones of inhibition produced by tetracycline were significantly smaller than those observed for the AgNPs. This enhanced activity of AgNPs is attributed to their multiple antibacterial mechanisms, which rely on effective cellular internalization. For *P. aeruginosa*, the low permeability of its Gram-negative outer membrane, attributed to specific functional groups, may limit interactions with AgNPs and reduce intracellular uptake, thereby minimizing oxidative and metabolic damage. The superior antibacterial performance of AgNPs over tetracycline is likely due to their ultra-small size and well-defined morphology, which allow greater surface contact, rapid internalization, and effective interaction with intracellular components. These effects are further sustained by the generation of ROS and release of Ag^+^ ions [13,14,55,56]. Additional details on the antibacterial mechanism of AgNPs will be discussed in the following section.

The antibacterial activity of the synthesized AgNPs was further analyzed by comparing their effects on Gram-positive and Gram-negative bacterial strains. The results revealed Gram-positive bacteria were generally more susceptible, with inhibition zones of 16, 17, and 15 mm recorded for *S. aureus*, MRSA, and *E. faecalis*, respectively. In comparison, *E. coli*, *K. pneumoniae*, *S. typhimurium*, and *P. aeruginosa* showed zones of 15, 15, 15, and 13 mm, indicating a consistent response among the Gram-negative strains, except for the more resistant *P. aeruginosa*. To statistically validate these observations, one-way ANOVA was performed. A significant difference (*p* < 0.05) was found among the inhibition zones of the Gram-positive strains, suggesting that their susceptibility to AgNPs may be influenced by differences in surface structures or functional groups. Conversely, no significant difference (*p* > 0.05) was found among the Gram-negative strains (excluding *P. aeruginosa*), implying a relatively uniform interaction with AgNPs, likely due to similar outer membrane properties.

To assess the dose-dependent antibacterial activity of AgNPs, concentrations of 100, 150, 200, and 250 µg/mL were tested against all bacterial strains. As shown in Figure 5 and Table 1, the inhibition zone diameters exhibited minimal variation across concentrations. For *S. aureus*, the zone remained unchanged at 100 and 150 µg/mL, slightly decreased at 200 µg/mL, and returned to the initial size at 250 µg/mL, suggesting a saturation effect where increasing concentration did not enhance antibacterial efficacy. This diminishing effect at higher concentrations is likely due to nanoparticle aggregation, where close proximity of excess AgNPs promotes cluster formation, reducing their interaction with and penetration into bacterial cells due to increased particle size. A similar trend was observed with MRSA, where all concentrations produced a consistent 17 mm zone of inhibition, reinforcing the saturation behavior. For *E. faecalis*, the zone increased from 15 mm at 100 µg/mL to 17 mm at 150 µg/mL, then plateaued, possibly due to its larger cell size enabling more initial AgNPs attachment and uptake, followed by saturation. One-way ANOVA analysis for *S. aureus*, MRSA, and *E. faecalis* confirmed no statistically significant differences (*p* > 0.05) among the inhibition ones at varying concentrations, indicating that increasing the dose did not meaningfully improve antibacterial performance. Thus, 100 µg/mL was adequate for effective inhibition of Gram-positive bacteria. For Gram-negative strains, the inhibition zones also remained largely unchanged or slightly decreased with increasing concentrations.

One-way ANOVA results (*p* > 0.05) further supported the absence of significant difference in antibacterial activity against *E. coli*, *K. pneumoniae*, *S. typhimurium*, and *P. aeruginosa* across the tested doses. The saturation effect observed at higher AgNPs concentrations reflects the interplay between dose, aggregation, and antibacterial efficacy. Above 100–150 µg/mL, increased aggregation reduces the surface area and limits nanoparticles’ interaction with bacteria, resulting in no significant increase in inhibition zones. This effect is further enhanced in physiological media, where salts and biomolecules promote aggregation and reduce AgNPs’ stability and antibacterial activity.

In the agar well diffusion assay, AgNPs produced inhibition zones comparable to or larger than those of tetracycline, likely due to the higher AgNP concentrations used. To substantiate these findings, IC_50_ values were determined from dose–response absorbance data plotted against varying concentrations of AgNPs and tetracycline. As shown in Table 2, tetracycline exhibited lower IC_50_ values (14.7–23 µg/mL) than AgNPs (33–46.1 µg/mL), confirming its greater potency while indicating notable antibacterial activity of AgNPs. Among the tested strains, *E. faecalis* was most sensitive to AgNPs (IC_50_ = 33 µg/mL), followed by MRSA (35 µg/mL) and *S. aureus* (IC_50_ = 42.3 µg/mL). The IC_50_ results aligned with the agar diffusion data, showing generally lower values for Gram-positive bacteria than for Gram-negative bacteria. Overall, although AgNPs were less potent than tetracycline, the difference was not substantial, highlighting their potential as complementary antimicrobial agents. Subsequently, MIC values of AgNPs were also determined to assess their antimicrobial strength.

**Figure 6:**
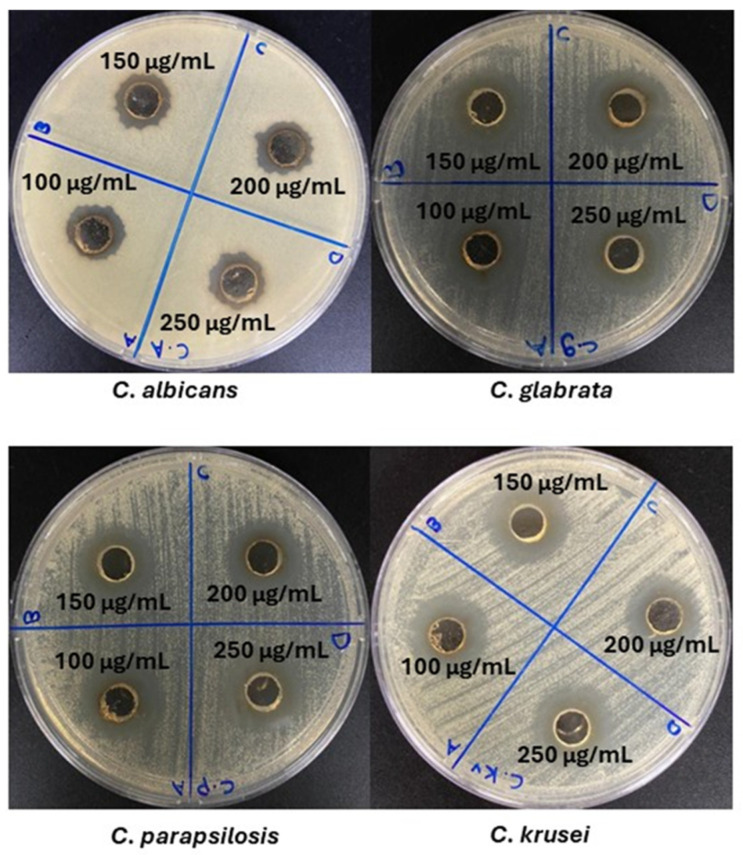
Agar well diffusion results showing the antifungal activity of AgNPs at different concentrations against *C. albicans*, *C. glabrata*, *C. parapsilosis*, and *C. krusei*.

The antifungal efficacy of the synthesized AgNPs was evaluated against *C. albicans*, *C. glabrata*, *C. parapsilosis*, and *C. krusei* using the agar well diffusion assay. Their activity was compared with that of the aqueous extract of *Celtis africana* leaves, AgNO_3_, and the standard antifungal agent fluconazole, all tested at a concentration of 100 µg/mL. As illustrated in Figure 6, the AgNPs produced distinct and strong zones of inhibition against all four fungal species, with measured diameters presented in Table 3. Consistent with antibacterial results, the plant extract showed no antifungal activity, while AgNO_3_ exhibited only moderate inhibition, in agreement with previous literature. Fluconazole demonstrated varying levels of activity, with inhibition zones ranging from 14 mm (*C. krusei*) to 20 mm (*C. albicans*). In contrast, the AgNPs displayed consistent antifungal activity across all tested fungi, with inhibition zones ranging between 19 and 21 mm, suggesting broad and stable efficacy regardless of fungal species. Interestingly, the inhibition zones produced by AgNPs against fungal strains were generally larger than those observed against bacteria. This could be attributed to the porous structure of fungal cell walls, rich in chitin and β-glucans, which may facilitate AgNPs penetration and enhance their antifungal effects. One-way ANOVA analysis confirmed no significant differences (*p* > 0.05) in the antifungal activity of AgNPs among the four *Candida* species. However, fluconazole exhibited significant variation (*p* < 0.05) across the same strains. These findings highlight the consistent and potent antifungal properties of AgNPs compared to the variability observed with standard drugs, underscoring their potential as a broad-spectrum antifungal agent.

The dose-dependent antifungal activity of AgNPs was evaluated, and the results are presented in Figure 6 and Table 3. Similarly to the antibacterial findings, increasing the concentration of AgNPs from 100 µg/mL to 250 µg/mL did not lead to a noticeable enhancement in the inhibition zone diameters. For instance, against *C. albicans*, the inhibition zones remained unchanged at 100 and 150 µg/mL, showed a slight decrease at 200 µg/mL, and then increased again at 250 µg/mL. In the case of *C. glabrata*, a sharp increase in the zones was observed at 150 µg/mL compared to 100 µg/mL, but it declined at higher concentrations. For *C. parapsilosis*, the inhibition zones remained consistent across all tested concentrations. Meanwhile, for *C. krusei*, the zone diameters decreased at 150 and 200 µg/mL but increased again at 250 µg/mL to match those observed at 100 µg/mL. To determine whether these variations were statistically significant, a one-way ANOVA was conducted. The analysis revealed no significant differences (*p* > 0.05) among the means across all concentrations for each fungal strain. This suggests that increasing the concentration of AgNPs beyond 100 µg/mL does not improve antifungal efficacy, likely due to a saturation effect. At higher concentrations, enhanced aggregation reduces the available surface area and limits effective interaction with fungal cells, thereby diminishing further improvements in antifungal activity. Given the potential effectiveness of concentrations below 100 µg/mL, the minimum inhibitory concentration (MIC) was determined to establish the lowest dose required for antimicrobial inhibition.

Table 4 compares the antimicrobial activity of AgNPs synthesized from *Celtis africana* with those from *Artemisia vulgaris*, *Moringa oleifera*, and *Nigella sativa*. The tested microbes included bacterial strains (*S. aureus*, MRSA, *E. faecalis*, *E. coli*, *K. pneumoniae*, *S. typhimurium*, and *P. aeruginosa*) and fungal species (*C. albicans*, *C. glabrata*, *C. parapsilosis*, and *C. krusei*). *Celtis africana*-based AgNPs showed broad-spectrum activity: strong inhibition (16–20 mm) against *S. aureus*, MRSA, and *E. faecalis*; moderate (11–15 mm) against *E. coli*, *K. pneumoniae*, *S. typhimurium*, *P. aeruginosa*, and *C. albicans*; and very strong (≥20 mm) against *C. glabrata*, *C. parapsilosis*, and *C. krusei*. *Artemisia vulgaris* exhibited generally lower activity, with gaps in data for *E. faecalis* and *C. krusei* [4,5]. For Moringa oleifera, data on several pathogens were unavailable, but reported activity was slightly higher than that of *Celtis africana* for others [7,8]. *Nigella sativa* showed comparable activity, though data were missing for some fungi and *K. pneumoniae* [10,11]. Overall, *Celtis africana*-based AgNPs demonstrated consistent and potent antimicrobial effects, often matching or surpassing those from the other plant sources.

### 3.9. Determination of MIC and MBC of AgNPs

**Figure 7:**
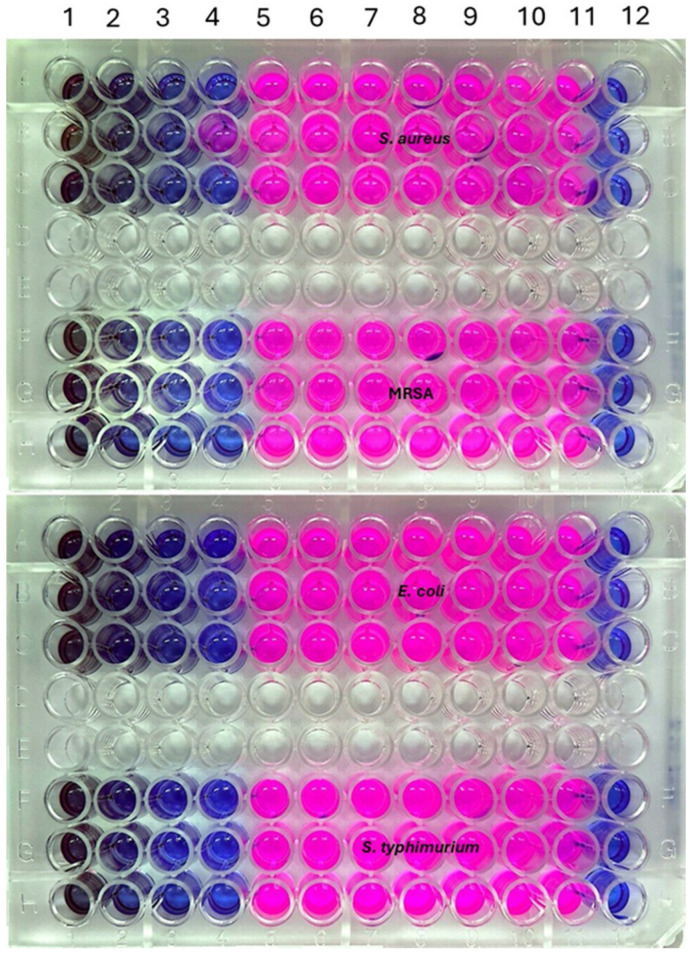
MIC determination of AgNPs against *S. aureus*, MRSA, *E. coli*, and *S. typhimurium* using the microdilution method.

**Figure 8:**
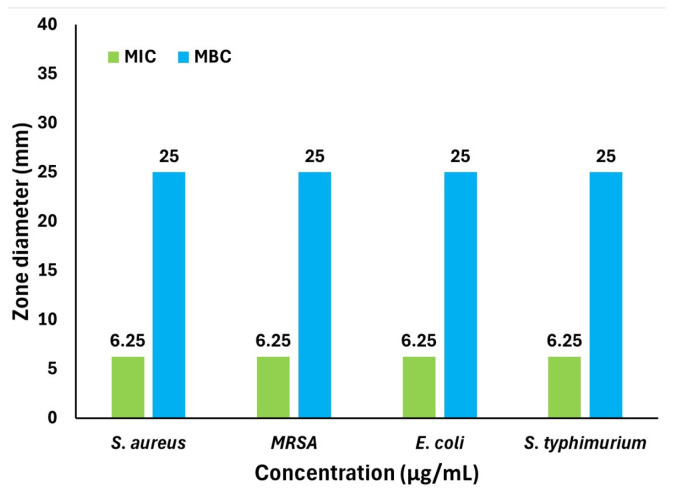
MIC and MBC values of AgNPs against *S. aureus*, MRSA, *E. coli*, and *S. typhimurium*.

The MIC refers to the lowest concentration of AgNPs that visibly inhibits bacterial growth [13,14,56]. To determine the MIC, two Gram-positive bacteria (*S. aureus* and MRSA) and two Gram-negative bacteria (*E. coli* and *S. typhimurium*) were selected. AgNPs were serially diluted using a two-fold dilution method to obtain concentrations of 100, 50, 25, 12.5, 6.25, 3.12, 1.56, 0.78, 0.39, and 0.195 µg/mL. The microdilution assay, performed in 96-well microtiter plates with resazurin dye, allowed for straightforward detection of bacterial inhibition. Resazurin retains a blue or purple color in the absence of bacterial growth and turns pink when growth occurs, enabling visual differentiation. The microtiter plate results for the four bacterial strains are shown in Figure 7, and the corresponding MIC values are illustrated as a bar diagram in Figure 8. Notably, AgNPs exhibited a consistent MIC value of 6.25 µg/mL across all tested strains. These findings are in agreement with the earlier antibacterial results, where 100 µg/mL of AgNPs produced slightly larger inhibition zones for *S. aures* and MRSA compared to *E. coli* and *S. typhimurium*. One-way ANOVA revealed no statistically significant difference in MIC values among the four strains (*p* > 0.05).

The minimum bactericidal concentration (MBC), the concentration required to kill 99% of bacteria, was also determined. Aliquots from wells showing no color change (indicating no bacterial growth) were spread on agar plates and incubated at 37 °C for 24 h. The lowest concentration that resulted in no visible colony formation was recorded as the MBC. Interestingly, all four strains shared an identical MBC value of 25 µg/mL, demonstrating the strong bactericidal effects of AgNPs at a concentration of four times the MIC. Overall, AgNPs synthesized using *Celtis africana* aqueous leaf extract exhibited potent and uniform antimicrobial activity, with a consistent MIC of 6.25 µg/mL and MBC of 25 µg/mL, values notably lower than those reported for many other plant-derived AgNPs (Table 5) [4,5,6,7,8,9,10,11,12].

### 3.10. Bacterial Growth Kinetics

**Figure 9:**
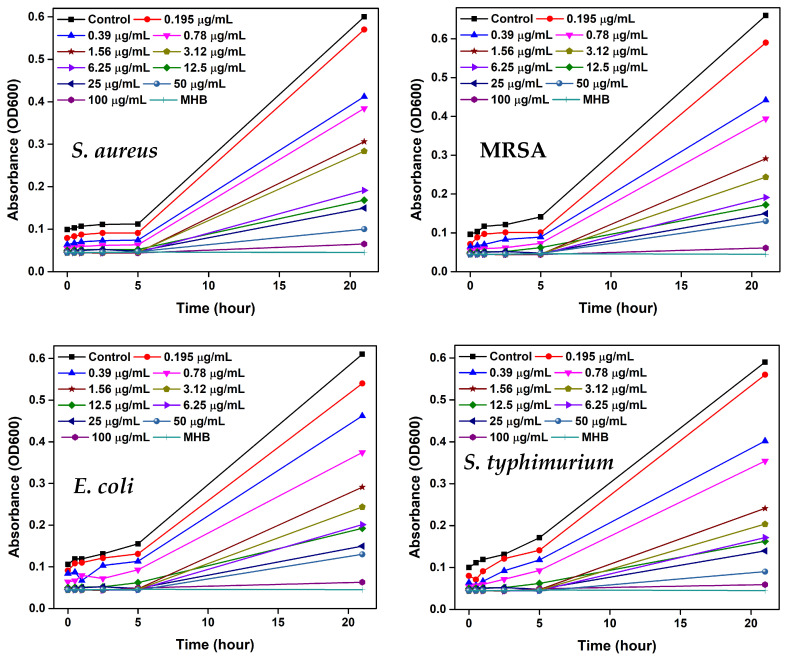
Growth curves analysis for *S. aureus*, MRSA, *E. coli*, and *S. typhimurium* treated with various concentrations of AgNPs over time.

Bacterial growth curves for *S. aureus*, MRSA, *E. coli*, and *S. typhimurium* were recorded over time and are shown in Figure 9. Absorbance at 600 nm was measured using a 96-well microtiter plate to monitor bacteria growth at AgNPs concentrations identical to those used in the MIC assay, 100, 50, 25, 12.5, 6.25, 3.12, 1.56, 0.78, 0.39, and 0.195 µg/mL. The data revealed that lower AgNPs concentrations failed to effectively suppress bacterial proliferation. However, as the concentration increased, growth was progressively inhibited, ultimately resulting in flattened curves. Complete inhibition was observed at 25 µg/mL for all strains, four times the MIC, indicating a strong bactericidal effect at this level. Interestingly, antibacterial efficacy declined at concentrations above 25 µg/mL, as evidenced by renewed bacterial growth. This may be due to nanoparticle aggregation over time at higher concentrations, reducing surface area, reactivity, and interaction with bacterial cells, thereby weakening their antimicrobial potential.

Moreover, the growth kinetics demonstrated a concentration-dependent shift in AgNPs activity [13,14,56]. At lower doses, AgNPs exhibited bacteriostatic effects, slowing bacterial growth without causing cell death. As the concentration increased, their activity transitioned to bactericidal, completely killing bacterial cells. Beyond this point, however, efficacy diminished, likely due to aggregation. These findings confirm that 25 µg/mL is the optimal concentration at which AgNPs function as a potent bactericidal against all tested bacterial strains.

### 3.11. Proposed Antimicrobial Mechanism of AgNPs

The antimicrobial mechanism of AgNPs is well-established in the literature and involves multiple coordinated actions [13,14,55,56]. It begins with the physical interaction of AgNPs with microbial cell surfaces, a process influenced by nanoparticle size, shape, and surface chemistry, particularly the phytochemical capping agents. Smaller, spherical AgNPs, like those synthesized in this study, provide a greater surface area for effective contact. Functional groups from phytochemicals (e.g., oxygen-containing groups and amide moieties such as (-CONH-) facilitate electrostatic and hydrogen bonding interactions with membrane components such as sulfate and phosphate groups, anchoring the nanoparticles near the cell surface.

Following attachment, AgNPs disrupt the cell wall and penetrate the interior, where they interface with vital intracellular functions. They bind to ribosomes and DNA, inhibiting protein synthesis and replication. Concurrently, they induce the generation of ROS, including superoxide anions and hydroxyl radicals, leading to oxidative damage to cellular components. The release of Ag^+^ ions further enhances antimicrobial activity by targeting membrane proteins and enzymes, disrupting respiration and metabolic pathways. Together, these mechanisms, membrane interactions, intracellular interference, oxidative stress, and ion release, account for the potent antimicrobial action of AgNPs. Previous studies have elucidated the multifaceted antimicrobial mechanisms of AgNPs, including membrane disruption, generation of reactive oxygen species, and interference with intracellular components [13,14,56].

### 3.12. Cytotoxic Potential of AgNPs from Celtis africana

To evaluate the cytotoxic potential of AgNPs, a concentration of 6.25 µg/mL, equivalent to MIC, was administered to HSF and MCF-7 cell lines. The cells were incubated for 24 h, and viability was assessed using the MTT assay. Untreated cells served as the positive control to establish baseline viability. Post-treatment analysis revealed distinct cellular responses between the two cell lines. HSF cells maintained high viability (93%), indicating minimal cytotoxicity, with only 7% of cells undergoing apoptosis. In contrast, MCF-7 cells exhibited a significant reduction in viability to 47.20%, with 52.80% of cells showing apoptotic features. These findings suggest that AgNPs selectively exert cytotoxic effects on cancerous cells while sparing normal fibroblasts [30].

**Figure 10:**
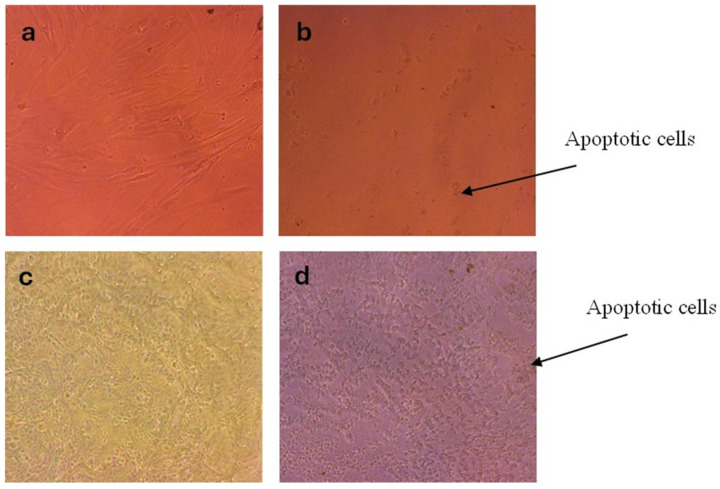
Morphological changes in HSF and MCF-7 cells after treatment with AgNPs for 24 h. (**a**) HSF control, (**b**) AgNPs-treated HSF, (**c**) MCF-7 control, and (**d**) AgNPs-treated MCF-7.

Microscopic observations using an inverted light microscope (Figure 10a–d) further supported these results. Treated MCF-7 cells displayed pronounced morphological changes, including cell shrinkage, membrane blebbing, and loss of their characteristic shape, hallmarks of apoptosis. Conversely, treated HSF cells retained their typical spindle-shaped morphology with minimal apoptotic alterations, indicating preserved cellular integrity and resistance to AgNPs-induced stress. Overall, these observations underscore the potential of AgNPs as a selective anticancer agent capable of inducing apoptosis in malignant cells while maintaining the viability of normal cells.

### 3.13. Antioxidant Activity

**Figure 11:**
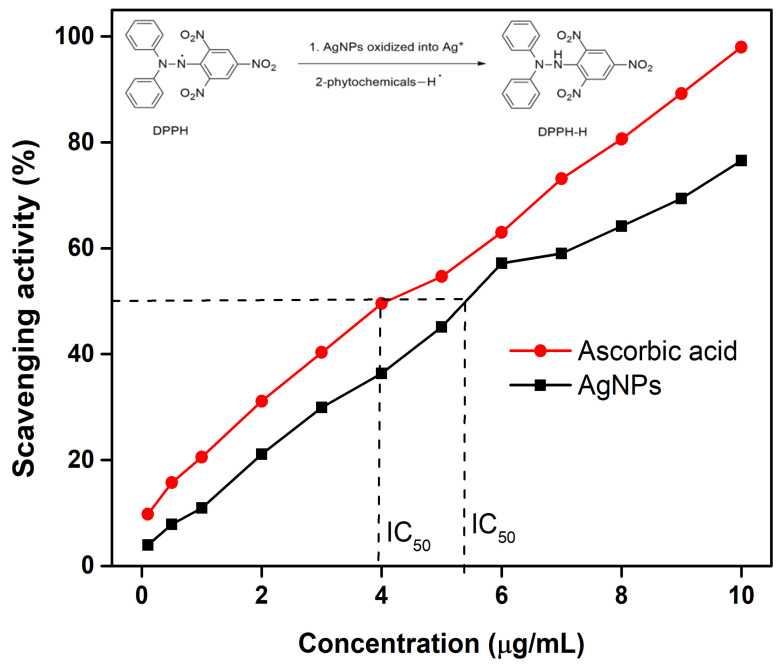
Dose-dependent DPPH scavenging activity of AgNPs and ascorbic acid, with inset showing the proposed radical neutralization mechanism.

The antioxidant potential of the synthesized AgNPs was evaluated using the DPPH radical scavenging assay, a simple and effective method for assessing radical-neutralizing capacity [13,14,56]. DPPH, a purple-colored radical, becomes colorless upon reduction by an antioxidant, indicating scavenging activity. The DPPH concentration was fixed at 10 µg/mL, while AgNPs concentrations ranged from 1 to 10 µg/mL. Absorbance was measured at 517 nm, and for comparison, ascorbic acid was tested under the same conditions. As shown in Figure 11, both AgNPs and ascorbic acid exhibited a concentration-dependent increase in scavenging activity. At 10 µg/mL, AgNPs achieved ~80% scavenging, while ascorbic acid reached 99%. Though slightly less potent, AgNPs still demonstrated strong antioxidant activity. IC_50_ values, derived from the dose–response curves by extrapolation, were 5.41 µg/mL for AgNPs and 3.94 µg/mL for ascorbic acid, indicating comparable antioxidant activity. Notably, the IC_50_ value of AgNPs synthesized from *Celtis africana* was lower than those reported for AgNPs derived from *Artemisia vulgaris*, *Moringa oleifera*, and *Nigella sativa* (Table 5), highlighting their excellent antioxidant potential. The antioxidant mechanism of AgNPs is attributed to two pathways: (1) electron donation by AgNPs, reducing DPPH to a non-radical form, and (2) hydrogen atom transfer from phytochemical capping agents, neutralizing the radical. An illustration of these mechanisms is provided in the inset of Figure 11.

### 3.14. Catalytic Activity of AgNPs

**Figure 12:**
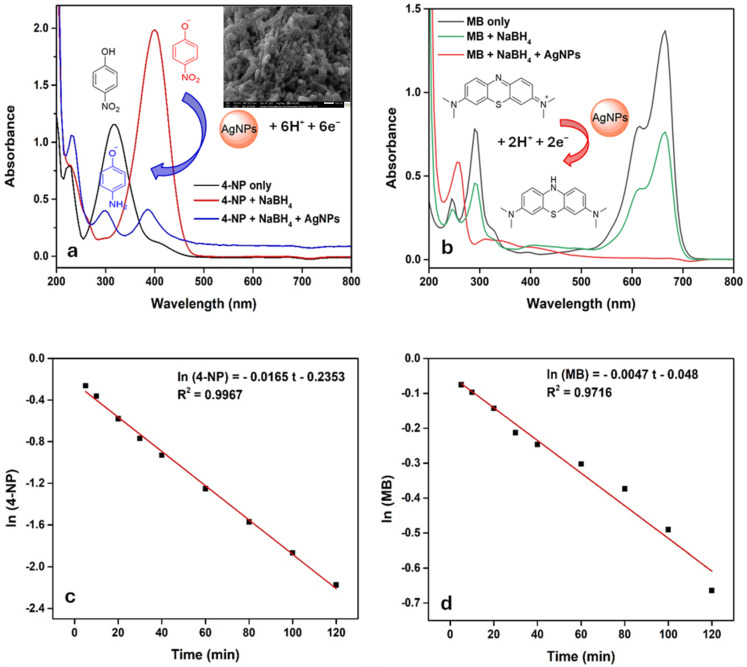
UV–Visible spectra of 4-NP (**a**) and MB (**b**) reduction by AgNPs and NaBH_4_, and corresponding concentrations change over time (**c**,**d**).

The catalytic efficiency of the synthesized AgNPs was assessed for the reduction of 4-NP and MB, two model pollutants. Reactions were monitored by UV–Visible spectroscopy (200–800 nm) and supported by observable color changes. The results are presented in Figure 12a,b. 4-NP appeared yellow, turning dark yellow/brown upon NaBH_4_ addition due to the formation of 4-nitrophenolate ions. However, without AgNPs, no significant reduction of 4-aminophenol (4-AP) was observed even after 3 h, as indicated by a persistent peak at 408 nm (shifted from 319 nm). In contrast, the introduction of AgNPs led to a rapid decrease in the 408 nm peak and a visible color change to colorless, confirming the complete reduction of 4-NP to 4-AP and highlighting the excellent catalytic potential of AgNPs.

Furthermore, AgNPs were recovered after the reduction of 4-NP and examined using SEM imaging, as shown in the inset of Figure 12a. The AgNPs underwent a pronounced morphological transformation, shifting from individually dispersed particles to aggregated, chain-like assemblies. This structural evolution is primarily driven by thermodynamic factors such as surface energy minimization and the possible desorption or degradation of stabilizing ligands during the catalytic process. Additionally, the reaction environment can induce changes in surface charge and polarity, promoting interparticle attraction and oriented attachment. Aggregation of AgNPs exerts a dual effect on catalytic performance [57]. On one hand, interconnected networks enhance electron transfer and create catalytic “hot spots,” accelerating kinetics. On the other hand, excessive aggregation tends to improve activity through synergistic interactions; however, this phenomenon requires further investigation and will be addressed in future studies.

MB reduction with NaBH_4_ alone showed only a gradual decrease in absorbance over time, with the solution remaining visibly blue. Upon AgNPs addition, the absorbance due to n-π* dropped sharply, accompanied by rapid decolorization, indicating successful reduction of B to its lecu-form [13,14,56]. In both cases, NaBH_4_ served as the reducing agent and 4-NP/MB as the electrophilic substrates. AgNPs facilitated electron transfer by offering an active surface, accelerating reduction [13,14,55]. These mechanisms are summarized in the insets of Figure 12a,b.

The rate constants for the reduction of 4-NP and MB were also determined by monitoring their concentration over time under excess NaBH_4_, ensuring pseudo-first order kinetics. As shown in Figure 12c,d, the reactions followed linear trends, with the regression equations ln(4-NP) = −0.0165 t − 0.2353 (R^2^ = 0.9967) and ln (MB) = −0.0047 t − 0.048 (R^2^ = 0.9716), from which the rate constants were calculated as 0.0165 s^−1^ for 4-NP and 0.0047 s^−1^ for MB. Compared to literature values (Table 5), the rate constant for 4-NP reduction was higher than those reported for AgNPs synthesized from *Artemisia vulgaris* (0.0121 s^−1^) and *Nigella sativa* (0.00152 s^−1^), indicating enhanced catalytic efficiency [6,9,12]. For MB reduction, the rate constant for *Moringa oleifera*-based AgNPs was 0.00438 s^−1^, while no specific values were found for *Artemisia vulgaris* and *Nigella sativa* [6,9,12]. These findings suggest that AgNPs synthesized using *Celtis africana* leaf extract possess superior catalytic activity and hold significant promise as sustainable nanocatalysts for environmental remediation applications.

### 3.15. Catalytic Activity of AgNPs in Industrial Wastewater

The catalytic activity of AgNPs for the reduction of 4-NP or MB in real water samples was assessed using industrial wastewater collected from the Jeddah Industrial area, South of Jeddah, Saudi Arabia. The sample was filtered (0.45 µM) and stored at ambient conditions, with a measured pH of approximately 8. Initial UV–Visible spectroscopy (200−1000 nm) revealed high background absorbance and overlapping signals, preventing clear detection of 4-NP or MB due to matrix complexity and low analyte concentrations.

**Figure 13:**
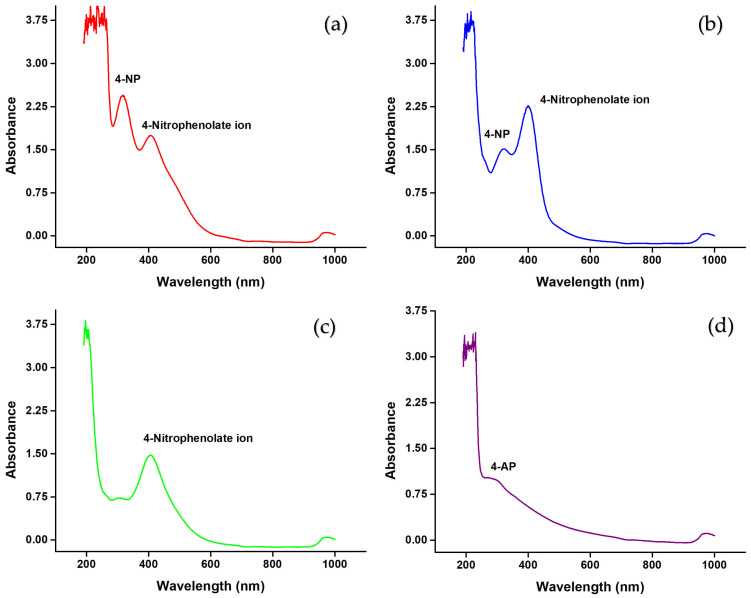
UV–Visible spectra showing (**a**) 4-NP after spiking 20 µg/mL, (**b**) after addition of AgNPs and NaBH_4_, (**c**) complete conversion of 4-NP into 4-nitrophenolate ions, and (**d**) reduction of 4-nitrophenolate ions into 4-AP.

To enable catalytic reduction studies, the sample was spiked with 20 µg/mL of 4-NP, and AgNPs and NaBH_4_ were added at concentrations consistent with Milli-Q water experiments. After spiking, a distinct 4-NP peak at 315 nm and 4-nitrophenolate ions at 406 nm were observed, even before the addition of AgNPs and NaBH_4_ (Figure 13a), attributable to the slightly basic pH promoting deprotonation of 4-NP. Upon addition of AgNPs and NaBH_4_, a decrease in the absorption of 4-NP and an increase in the absorption of 4-nitrophenolate ions were observed (Figure 13b). Compared to Milli-Q water, the conversion of 4-NP to 4-nitrophenolate ions in wastewater was slower, requiring about 10–20 min for complete conversion (Figure 13c). The subsequent reduction of 4-nitrophenolate ions to 4-AP was also substantially slower; while nearly instantaneous in Milli-Q water, it took approximately 2–3 h in the real water samples (Figure 13d). Despite matrix effects and the complex nature of the wastewater, AgNPs synthesized using *Celtis africana* leaf extract were capable of completely reducing 4-NP, underscoring their potential as effective green catalysts for environmental remediation.

## 4. Conclusions

This study successfully demonstrated the phytogenic synthesis of AgNPs using the leaf extract of *Celtis africana*, a rare species in Saudi Arabia. Characterization confirmed the formation of ultra-small, spherical AgNPs with strong SPR and an fcc crystalline structure. UV–Visible and FTIR analysis suggested the involvement of phenolic amide compounds, while HPLC confirmed the presence of caffeoyltyramine in the aqueous extract. The oxidative transformation of these phytochemicals likely resulted in the capping of AgNPs with aromatic residues, contributing to their colloidal stability. The synthesized AgNPs exhibited potential antimicrobial activity against a broad spectrum of Gram-positive (*S. aureus*, MRSA, and *E. faecalis*) and Gram-negative (*E. coli*, *K. pneumoniae*, *S. typhimurium*, and *P. aeruginosa*) and clinically relevant fungal species *C. albicans*, *C. glabrata*, *C. parapsilosis*, and *C. krusei*. Notably, low MICs and concentration-dependent growth inhibition highlighted their strong bactericidal and fungicidal potential. The anticancer properties of the synthesized AgNPs were also confirmed against MCF-7 cells, while HSF were minimally affected. Antioxidant activity, assessed via DPPH radical scavenging, showed a linear and comparable performance to ascorbic acid, with an IC_50_ of 6 µg/mL. Additionally, the AgNPs demonstrated rapid and efficient catalytic reduction of 4-NP and MB in both Milli-Q and wastewater, indicating promising environmental remediation capabilities. Overall, the findings confirm the multifunctional potential of *Celtis africana*-derived AgNPs for biomedical and environmental applications. Future investigations should explore their cytotoxicity and biocompatibility to support further development in clinical and ecological contexts.

## Figures and Tables

**Table 1:** Comparison of antibacterial activity of AgNPs with aqueous extract of *Celtis africana*, AgNO_3,_ and tetracycline.

Microorganism	Aqueous Extract	AgNPs				AgNO_3_	Tetracycline
		µg/mL					
		100	150	200	250	100	40
*S. aureus*	-	16 ± 0.13 ^a^	16 ± 0.12 ^a^	15 ± 0.12 ^c^	16 ± 0.11 ^a^	11 ± 0.12 ^h^	13 ± 0.12 ^d^
MRSA	-	17 ± 0.1 ^b^	17 ± 0.1 ^b^	17 ± 0.11 ^b^	17 ± 0.12 ^b^	12 ± 0.12 ^f^	16 ± 0.11 ^a^
*E. faecalis*	-	15 ± 0.13 ^c^	17 ± 0.11 ^b^	17 ± 0.11 ^b^	17 ± 0.11 ^b^	11 ± 0.11 ^h^	12 ± 0.13 ^f^
*E. coli*	-	15 ± 0.11 ^c^	15 ± 0.12 ^c^	14 ± 0.12 ^e^	14 ± 0.13 ^e^	13 ± 0.13 ^d^	10 ± 0.11 ^g^
*K. pneumoniae*	-	15 ± 0.11 ^c^	15 ± 0.12 ^c^	14 ± 0.11 ^e^	14 ± 0.14 ^e^	12 ± 0.13 ^f^	14 ± 0.14 ^e^
*S. typhimurium*	-	15 ± 0.11 ^c^	15 ± 0.11 ^c^	14 ± 0.11 ^e^	15 ± 0.11 ^c^	12 ± 0.12 ^f^	16 ± 0.13 ^a^
*P. aeruginosa*	-	13 ± 0.11 ^d^	13 ± 0.11 ^d^	13 ± 0.11 ^d^	12 ± 0.11 ^f^	12 ± 0.12 ^f^	17 ± 0.11 ^b^

Values with different superscript letters (a–h) differ significantly at *p* < 0.05, while values sharing the same letter are not significantly different (*p* > 0.05).

**Table 2:** Comparison of IC_50_ value for AgNPs and tetracycline against tested bacterial strains, determined based on dose-responsive behavior.

Microorganism	AgNPs	Tetracycline
	IC_50_ (µg/mL)	
*S. aureus*	42.3	19.1
MRSA	35	16.42
*E. faecalis*	33	21.2
*E. coli*	40	23
*K. pneumoniae*	41	18.4
*S. typhimurium*	37.8	15.9
*P. aeruginosa*	46.1	14.7

**Table 3:** Comparison of antifungal activity of AgNPs with aqueous extract of *Celtis africana*, AgNO_3,_ and fluconazole.

Microorganism	Aqueous Extract	AgNPs				AgNO_3_	Fluconazole
		µg/mL					
		100	150	200	250	100	25
*C. albicans*	-	15 ± 0.11 ^a^	15 ± 0.11 ^a^	13 ± 0.11 ^c^	15 ± 0.12 ^a^	15 ± 0.11 ^a^	20 ± 0.12 ^b^
*C. glabrata*	-	20 ± 0.1 ^b^	24 ± 0.1 ^d^	20 ± 0.12 ^b^	21 ± 0.11 ^e^	14 ± 0.13 ^f^	19 ± 0.11 ^g^
*C. parapsilosis*	-	20 ± 0.11 ^b^	20 ± 0.12 ^b^	20 ± 0.11 ^b^	20 ± 0.11 ^b^	14 ± 0.11 ^f^	17 ± 0.13 ^h^
*C. krusei*	-	21 ± 0.11 ^e^	20 ± 0.11 ^b^	19 ± 0.11 ^g^	21 ± 0.13 ^e^	14 ± 0.11 ^f^	14 ± 0.11 ^f^

Values with different superscript letters (a–h) differ significantly at *p* < 0.05, while values sharing the same letter are not significantly different (*p* > 0.05).

**Table 4:** Antimicrobial activity of plant-derived AgNPs classified by inhibition zone (weak ≤ 10 mm; moderate 11–15 nm; strong 16–20 mm; very strong > 20 mm).

Microorganism	*Celtis africana*	*Artemisia vulgaris*	*Moringa oleifera*	*Nigella sativa*
*S. aureus*	Strong	strong	Strong	Strong
MRSA	Strong	Moderate	Moderate	strong
*E. faecalis*	Strong	Not specified	Not specified	Strong
*E. coli*	Moderate	Strong	Very strong	Moderate
*K. pneumoniae*	Moderate	Moderate	Moderate	Not specified
*S. typhimurium*	Moderate	Moderate	Not specified	Moderate
*P. aeruginosa*	Moderate	Moderate	Strong	Moderate
*C. albicans*	Moderate	strong	Moderate	Moderate
*C. glabrata*	Very strong	Not specified	Not specified	Not specified
*C. parapsilosis*	Very strong	Not specified	Not specified	Not specified
*C. krusei*	Very strong	Not specified	Not specified	Not specified

**Table 5:** Summary comparison of MIC, MBC, antioxidant activity (IC_50_), and rate constant for the reduction of 4-NP and MB using AgNPs synthesized from *Celtis africana*, *Artemisia vulgaris*, *Moringa oleifera*, and *Nigella sativa*.

Plant Source	AgNPs			
	MIC (µg/mL)	MBC (µg/mL)	Antioxidant (IC_50_, (µg/mL)	Rate Constant (s^−1^)
*Celtis Africana*	6.25	25	5.41	0.0165 (4-NP) 0.0047 (MB)
*Artemisia vulgaris* [4,5,6]	250	-	11.4	0.0121 (4-NP)
*Moringa oleifera* [7,8,9]	14–24	-	16.4	0.00438 (MB)
*Nigella sativa* [10,11,12]	15–30	50	-	0.00152 (4-NP) 0.007 (MB)

## Data Availability

The data supporting the findings of this study are available from the corresponding author upon reasonable request.
